# Magnetic Resonance Imaging of Ischemia Viability Thresholds and the Neurovascular Unit

**DOI:** 10.3390/s130606981

**Published:** 2013-05-27

**Authors:** Philip A. Barber

**Affiliations:** 1 Departments of Clinical Neurosciences, University of Calgary, 3330 Hospital Drive NW, Calgary, ALB T2N 4N1, Canada; E-Mail: pabarber@ucalgary.ca; Tel.: +1-403-210-3881; Fax: +1-403-270-8928; 2 Departments of Radiology, University of Calgary, 3330 Hospital Drive NW, Calgary, ALB T2N 4N1, Canada; 3 Hotchkiss Brain Institute, University of Calgary, 3330 Hospital Drive NW, Calgary, ALB T2N 4N1, Canada

**Keywords:** stroke, ischemic penumbra, neurovascular unit, MRI

## Abstract

Neuroimaging has improved our understanding of the evolution of stroke at discreet time points helping to identify irreversibly damaged and potentially reversible ischemic brain. Neuroimaging has also contributed considerably to the basic premise of acute stroke therapy which is to salvage some portion of the ischemic region from evolving into infarction, and by doing so, maintaining brain function and improving outcome. The term neurovascular unit (NVU) broadens the concept of the ischemic penumbra by linking the microcirculation with neuronal-glial interactions during ischemia reperfusion. Strategies that attempt to preserve the individual components (endothelium, glia and neurons) of the NVU are unlikely to be helpful if blood flow is not fully restored to the microcirculation. Magnetic resonance imaging (MRI) is the foremost imaging technology able to bridge both basic science and the clinic via non-invasive real time high-resolution anatomical delineation of disease manifestations at the molecular and ionic level. Current MRI based technologies have focused on the mismatch between perfusion-weighted imaging (PWI) and diffusion weighted imaging (DWI) signals to estimate the tissue that could be saved if reperfusion was achieved. Future directions of MRI may focus on the discordance of recanalization and reperfusion, providing complimentary pathophysiological information to current compartmental paradigms of infarct core (DWI) and penumbra (PWI) with imaging information related to cerebral blood flow, BBB permeability, inflammation, and oedema formation in the early acute phase. In this review we outline advances in our understanding of stroke pathophysiology with imaging, transcending animal stroke models to human stroke, and describing the potential translation of MRI to image important interactions relevant to acute stroke at the interface of the neurovascular unit.

## 1. Introduction

Evolution of brain damage due to stroke is a highly complex process, involving cerebrovascular and parenchymal tissues through the interaction of multiple mechanisms. Neuroimaging has contributed considerably to the understanding the pathophysiology of stroke in living animals and humans, and has been the keystone to therapeutic advancement for stroke care [1]. The concept of the *ischemic penumbra* and *infarct core* in the early 1970s has transcended from a basic understanding of ischemic core and salveagable ischemic brain based on the observation of anoxic depolarization and energy metabolism [2]. Recently, this compartmentalized concept has incorporated important processes at the level of the microvasculature following cerebral ischemia. The ischemic penumbra implies the basic premise that acute stroke therapy can salvage some portion of the ischemic region from evolving into infarction and by doing so maintaining brain function and improving outcome [1].

The concept of the Neurovascular Unit (NVU) incorporates critical and metabolic tissue viability thresholds with cellular interactions involving endothelium, with astrocytes, and neurons with the blood brain barrier (BBB) and extracellular matrix. Under physiological conditions the BBB provides the critical, physical, metabolic, and neurological barrier that separates the CNS from the peripheral circulation [3]. Focal cerebral ischaemia, as is observed in acute stroke, is responsible for the loss of endothelial cell integrity resulting in an increase of vascular permeability [4]. The disruption of the BBB results in the formation of a vasogenic oedema, which causes further damage in the surrounding tissue, especially when associated with hemorrhagic transformation [5]. Several mediators may contribute to the stroke-induced alterations of the BBB: reactive oxygen species (ROS) [6,7], platelet activating factor, tumour necrosis factor-α [8], vascular endothelial growth factor [9], and matrix metalloproteinases [10].

Positron emission tomography (PET) translated the early concept of the penumbra by visualizing the relationship of cerebral blood flow changes to the metabolic demands of ischemic tissue. This initial step of translating electrophysiological observations to human imaging was followed by further iterations in the evolution of clinical imaging paradigms that has transformed the concept of the penumbra to one of “mismatch” between ischemic core and potentially salvageable tissue by clinically available technologies-MRI and computed tomography [11,12]. With the help of this imaging technology came refinements in selecting, for clinical trials, patients for therapeutic intervention for acute stroke with thrombolysis and endovascular therapy. Such advanced neuroimaging techniques have been studied in clinical trials [12–15].

Despite inclusion of increasingly sophisticated neuroimaging there has been a failure of numerous neuroprotective clinical trials. This has led to the realization that refinements to the imaging of ischemic injury is needed to visualize additional aspects of the complex process of stroke, that includes imaging assessment of cerebrovascular and parenchymal tissue. The introduction of the NVU offers a conceptual and practical approach, which fundamentally links the integrity of the microvessel with neuronal and glial interactions [16,17]. From a therapeutic perspective this unitary conceptual framework implies that there will be limited recovery of function unless blood flow is re-established and all parts of the unit recover because of the ischemic vulnerability of individual sensitive components of the unit [17,18]. Biological understanding of the processes of ischemia and the NVU has now developed through technological advancements in molecular biology and *in vivo* imaging including non-invasive imaging techniques. Recent work with MRI has evolved from imaging ischemic viability to include imaging of the microvasculature, specifically pathological processes resulting in BBB dysfunction, and concurrent molecular and cellular inflammatory events [19,20]. This review focuses on stroke related pathophysiological processes that can be currently imaged, describing the shortcomings of each. The unmet needs of currently available imaging modalities are identified that may be achieved in the future with more sophisticated molecular imaging.

## 2. Basic Mechanisms of Stroke

Acute cerebral ischemia occurs following mechanical occlusion of cerebral blood vessels, usually by embolus [21]. When blood flow to the brain is reduced survival of brain tissue depends on the intensity and duration of the ischemia and the availability of collateral blood flow. During moderate to severe cerebral ischemia, autoregulation is impaired. This has allowed investigators to reduce cerebral blood flow (CBF) and assess the critical flow thresholds for certain functions. At blood-flow levels around 20 mL/100 g/min, the oxygen extraction fraction (OEF) becomes maximal, the cerebral metabolic rate for oxygen (CMRO_2_) begins to fall [22]. Normal neuronal function of the cerebral cortex is affected, and cortical electroencephalographic activity ceases [23]. This degree of ischemia represents a viability threshold defined as the *loss of neuronal electrical function.* At levels below 10 mL/100 g/min, cell membranes and function are severely affected [24]. At this threshold, lack of oxygen inhibits the mitochondrial metabolism and activates the inefficient anaerobic metabolism of glucose causing a local rise in lactate production and so a fall in pH, leading to intra- and extracellular acidosis. The energy dependent function of the cell membrane to maintain ion homeostasis becomes progressively impaired. Potassium ions leak out of cells into the extracellular space, Na^+^ and water enter cells (cytotoxic oedema), and Ca^2+^ enters the cell, where it impairs mitochondrial function and compromises intracellular membranes to control subsequent ion fluxes, leading to further cytotoxicity. This degree of ischemia represents a *threshold of loss of cellular ion homeostasis*.

**Figure 1.**
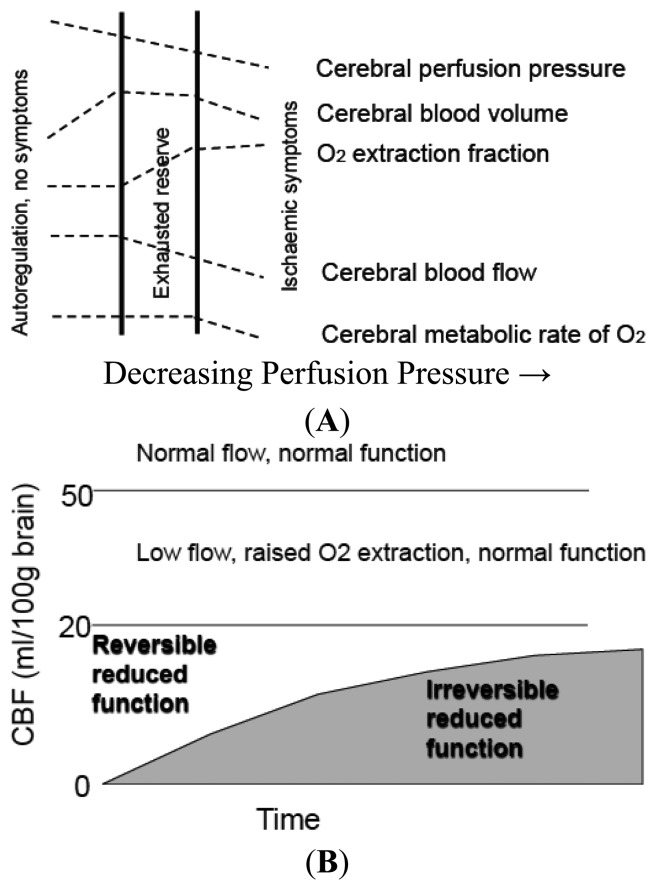
**(A)** Cerebral Blood Flow and Metabolic Thresholds. With falling Cerebral Perfusion Pressure (CPP) as occurs distal to a cerebral artery occlusion, intracranial arteries dilate to maintain CBF- a process termed autoregulation. This results in an increase in Cerebral Blood Volume. When vasodilation is maximal, further falls in CPP result in a fall in CBF and results in increase in Oxygen Extraction Fraction (OEF) to maintain tissue oxygenation. When OEF is maximal further falls in CPP lead to reduction in Cerebral Metabolic Rate for Oxygen utilization (CMRO2). **(B)** The combined effects of residual CBF and duration of ischemia on reversibility of neuronal dysfunction during focal ischemia. The gray shaded region outlines the limits of severity and duration of ischemia, distinguishing tissue “not at risk” from functionally impaired tissue. Schematics are drawn from concepts attributed to Baron and Heiss *et al* [25,26].

These two concepts of critical thresholds of electrical and membrane failure define upper and lower flow limits of the ischemic penumbra, a fundamental component of the concept being that penumbra tissue is reversibly injured [2,24] but this is dependent on both the severity and the duration of ischemia; with increasing time the infarct core grows into the penumbra (Figure 1) [25,26]. From a pragmatic perspective the penumbra is also characterized by a response to pharmacological agents [14]. However, recent studies of functional and metabolic disturbances suggest a more complex pattern of thresholds. During the initial few hours of vascular occlusion, different metabolic functions breakdown at varying CBF levels. At declining flow rates in both global and focal models of ischemia, protein synthesis is first inhibited in neurons, followed by anaerobic glycolysis, the release of neurotransmitters, impaired energy metabolism, and finally membrane depolarization [27]. Mechanisms that give rise to ischemic cell death occur via three major mediators: unregulated increases of Ca^2+^ concentration intracellularly, tissue acidosis, nitric oxide and free radical production. In the early phases of ischemia, the injury is compromised by waves of spreading depression that further compromise regional CBF. In the minutes, hours, days after this initial ischemic insult, brain injury is modulated by: inflammatory processes, the induction of immediate early genes, free radicals, and later by apoptotic mechanisms [28].

**Figure 2.**
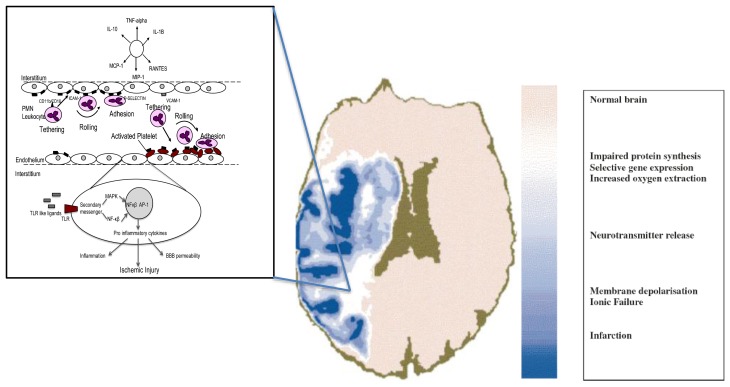
Two conceptual frameworks of Ischemic Stroke: the Penumbra and the Neurovascular Unit. The heterogeneity of the ischemic penumbra is illustrated. From the onset of focal ischemia the core and the penumbra are dynamic in space and time. A region of low perfusion in which cells have lost their membrane potential (core) is surrounded by an area in which intermediate perfusion prevails (penumbra) and cells depolarize intermittently. The CBF and metabolic viability thresholds (scale on right of figure) identify reversible and irreversibly injured tissue. The potentially reversible injury is modified by microvascular cellular and molecular responses at the level of the neurovascular unit (NVU, inset figure on left), mechanisms that include vasogenic oedema, BBB dysfunction and hemorrhagic transformation. During ischemia endothelial cells express PAR1, tissue factor (TF) and matrix metalloproteinases (MMPs). Together these facilitate the endothelial inflammatory response causing aggregation of platelets, fibrin degradation and leukocyte recruitment. These phenomenon potentially contribute to microvascular “no reflow”. Also leukocytes adherent to endothelium can cause endothelial dysfunction, transvascular protein leakage and oedema, leading to brain injury. MMPs contribute to the degradation of extracellular matrix causing an increase in BBB permeability. Endogenous ligands from damaged cells cause the expression of Toll Like Receptors (TLRs). Through complex signaling pathways pro-inflammatory cytokines are produced via transcription factors such as NFκβ and AP-1.

The microvasculature responds very quickly to the changes in CBF, and when critical thresholds are reached, endothelial cells rapidly convert into a pro-inflammatory and pro-thrombotic state by the up regulation of various humoral intermediaries, such as proteinase activated receptor 1 (PAR 1), endothelial tissue factor and matrix metalloproteinases (MMPs) [29,30] in the ischemic core and peunmbra which facilitate inflammation and BBB dysfunction [31-33]. This process facilitates the accumulation of fibrin, platelets and neutrophils, which results in micro vascular obstruction. Matrix metalloproteinases degrade the neurovascular matrix on the abluminal side causing acute BBB disruption. The release of endogenous ligands from damaged cells leads to the activation of Toll Like Receptors (TLRs). Their signaling causes several mediators to promote the production of pro-inflammatory cytokines via the activation of transcription factors such as NF-κβ and AP1, perpetuating a cycle of neurovascular damage (Figure 2) [34-36]. Endothelial cells facilitate selective leukocyte recruitment by a sequence of interactions with brain endothelial cell adhesion molecules [37,38], controlling leukocyte rolling, tethering, and adhesion along endothelial cells. Ultimately, leukocytes transmigrate from the luminal to the abluminal side of the endothelial layer. One consequence of these events is that microvessels become obstructed within the territory-at-risk, with focal loss of permeability barriers and changes in endothelium-astrocyte-neuron relationships. The obstruction is most prominent in end arteries, for instance, the microvasculature of the striatum [39,40]. Activated platelets and fibrin, caused by the generation of thrombin, are also inherently involved in the microvessel obstruction. The microvessel wall undergoes rapid and dynamic change affecting matrix integrity of the basal lamina and matrix receptors. The change is concomitant with neuronal injury. Also, at this time the expression of the matrix constituents (basal lamina, laminin, collagen IV, cellular fibronectin, and perlecan) decrease substantially [41,42]. It has also been established that endothelial and astrocyte cytoskeletal structures are compromised by a decrease in endothelial cell β_1_-integrin receptor and integrin α_6_β_4_ on astrocyte end-feet in the first hour following middle cerebral artery (MCA) occlusion [40-43].

## 3. Imaging Tissue Viability during Ischemia

The imaging surrogates of infarct core should reflect histological markers of irreversible cellular injury, unresponsive to increases of blood flow during reperfusion or dynamic changes of flow in and around the injury. They should also reflect what we understand from the pathophysiology that with time the core enlarges and the severity increases [43,44]. Markers to assess morphological integrity achieved in experimental studies are predicated on invasive procedures, and therefore such markers cannot be determined in humans [44]. There are several challenges for imaging penumbra in human beings: (1) most techniques used clinically do not provide information about tissue viability; and, (2) ischemia is a highly dynamic process and physiological variables can only be measured at a few discrete time points and repeated measures are extremely challenging in contrast with animal experiments.

The identification of penumbra necessitates measuring reduced CBF less than the functional threshold and biochemically differentiating morphologically viable from dead brain tissue. An early demonstration of the functional threshold was demonstrated in monkeys exposed to focal cerebral ischemia [45]. A reduction in blood flow following middle cerebral artery occlusion led to the developmental of a neurological deficit at flow rates of 23 mL/100 g of brain per minute, and if lowered further caused irreversible paralysis at blood flow rates of 8 mL/100 g per minute [45]. This observation confirmed the concept of CBF viability thresholds but considerable variability exists determined by complex interactions dependent on the functional threshold of individual neurons, age of subject, and brain location (grey *vs.* white matter) [46,47]. Biochemical substrate biomarkers show similar thresholds but the pattern is more complex and the CBF values fall within a wider range; with declining flow rates in the range of 15-35 mL/100 g of brain per minute, protein synthesis is inhibited followed by the preference for an aerobic glycolysis at 35 mL/100 g of brain per minute, followed by the release of neurotransmitters and the impairment of energy metabolism at around 20 mL/100 g of brain per minute. Finally, terminal depolarization and concomitant potassium influx is observed at 6–15 mL/100 g of brain per minute [27].

The use of positron emission tomography (PET) in human subjects has been aimed at identifying irreversibly damaged and ischemically compromised brain. PET utilizing 15-oxygen tracers are considered to provide the reference standard for the CBF measurements [9,48–50]. The imaging paradigm clinically has been a CBF measure combined with metabolic surrogates of tissue viability signaling permanent tissue destruction. PET using 15 oxygen tracers allows quantitative assessment of CBF, cerebral metabolic rate of oxygen (CMRO2), oxygen extraction (OEF) and cerebral blood volume (CBV), independently measuring perfusion and energy metabolism and demonstrating the uncoupling of each at discrete time intervals following ischemia (Figure 1). The core is defined by reduced CBF and CMRO2, and the penumbra as a region beyond this where CBF is reduced, but OEF is increased and CMRO2 is normal.

Understanding the limitations of a single cerebral blood flow threshold has evolved from PET imaging in focal ischemia and this knowledge base could be applied to more conventional clinical imaging paradigms like CT and MRI. Five main factors account individually to the variance of the PET acquired data: blood flow discrepancies between grey and white matter, age determinants, methodological variability related to blood flow between regions of interest (ROI), voxel-based analysis (VBA), and duration of ischemia as an independent predictor of tissue viability. Blood flow measurements are dynamic represented visually in the clinical scenario at variable time points without knowledge of CBF before or after. This contrasts dramatically with the relatively controlled environment of animal experiments; in humans the duration of ischemia cannot be controlled, nor the site of occlusion, nor the location of ischemia.

During ischemia OEF compensates for the ischemic challenge by increases of up to 80% compared to approximately 30% in the resting state [50]. This elevation of OEF along with reduction of CBF can discriminate the ischemic compartments of non-viable tissue *vs.* penumbra. The combination of OEF and CBF x arterial oxygen content is CMRO2. Data supports that CMRO2 is the preferred marker to delineate infarcted from viable tissue [50]. However, there are logistical challenges with obtaining OEF measurements, as the technique requires a steady-state inhalation approach, which makes it impractical for imaging stroke patients. However, alternative techniques have been successfully developed; these include ^11^C-flumazenil (FMZ) and ^18^F-fluoromidoianzol (F MISO). ^11^C-FMZ is a marker of cortical neuronal integrity and may be a reliable surrogate of ischemic core capable of delineating penumbra in combination of ^15^O-water PET [18,46,51,52]. F MIZO is a marker of hypoxic tissue that may allow direct visualization of hypoxia impairment [53].

There are other limitations with PET imaging. Many clinical studies recruited small numbers of subjects, providing data on blood flow, metabolic values, and final infarction [46,54–56]. These studies have shown that two variables are consistently correlated with respect to viability, namely contemporaneously measured CBF and CMRO2 utilization. However, the identification of CBF values corresponding to metabolic determinants of tissue viability values of infarcted *vs.* non-infarcted penumbra tissue is a matter of controversy; CBF values are dependent on the separation of cortex and white matter, measurement error at low trace levels and methodological differences between applied region of interest techniques *vs.* voxel-based analysis [50]. Given the PET limitations, preliminary MR methods have been developed to image OEF and CMRO2 in animals and humans [57–59].

## 4. MRI Perfusion/Diffusion “Mismatch”

**Figure 3.**
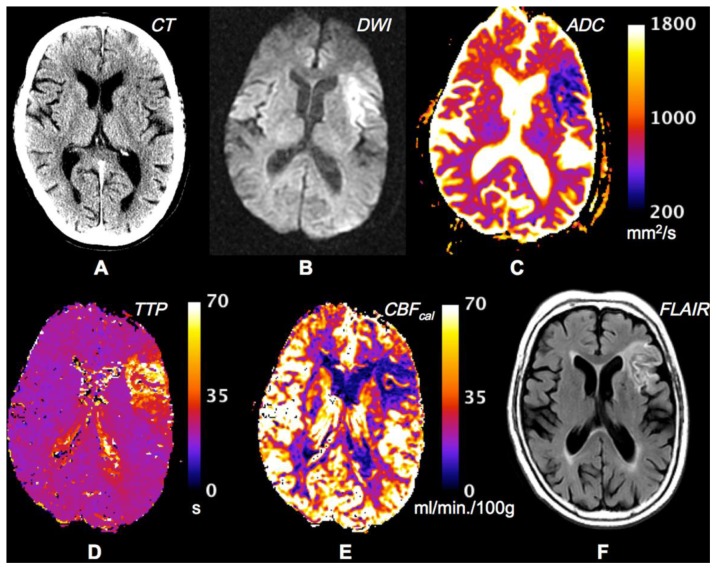
CT and MR imaging for an acute stroke patient (76-year-old female). **(A)** The CT image shows slight hypointensity in the left anterior insular ribbon, as indicated by the yellow arrow. The apparent loss of structural definition suggests ischemia in the middle cerebral artery (MCA) territory. **(B)** DWI image shows very hyperintense areas interpreted as a region of infarction. **(C)** ADC map shows decreased diffusion of water in absolute units within the markedly hyperintense DW region. **(D)** Time-to-peak (TTP) map calculated from a DSC perfusion sequence showing delayed flow to ischemic region. **(E)** Cerebral blood flow (CBF) map computed using deconvolution with an arterial input function as a reference, and then calibrated to white matter in the contralateral side to provide a semi-quantitative depiction of blood flow. **(F)** FLAIR image from day 30 follow-up MR exam shows final infarct to match closely with acute infarct. There was a delay time of 1.2 h from stroke onset to CT imaging, and an additional delay of 2.6 h to MR imaging. The patient was treated with intravenous tPA 1.5 h after onset–before the MR exam. DWI typically depicts infarction with greater lesion contrast as is the case here. The lack of a distinct perfusion-diffusion mismatch and no evident growth on follow- up may be at least partly attributable to recanalization occurring as a result of tPA administration (courtesy of Robert K Kosior, University of Calgary).

**Figure 4.**
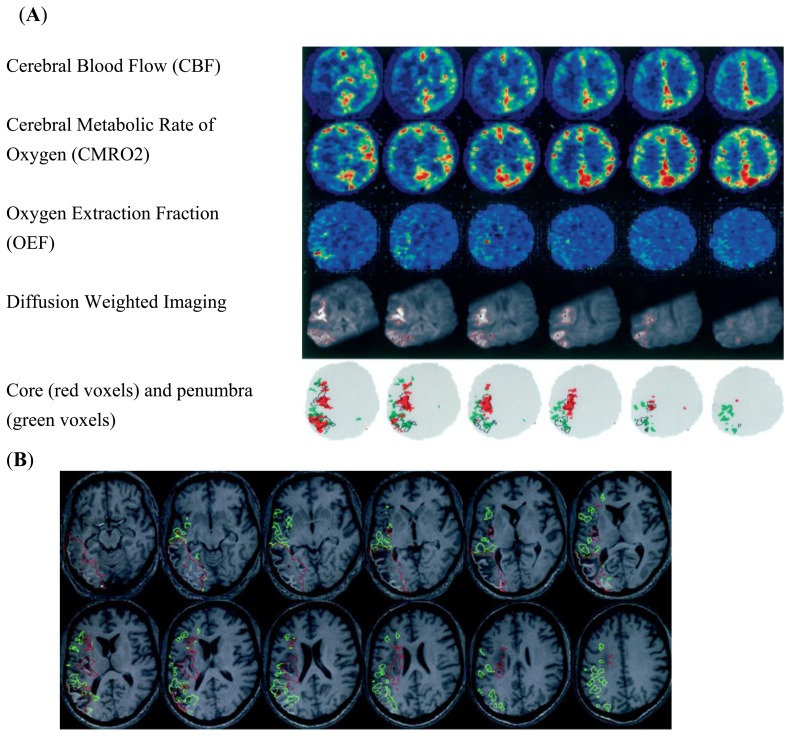
Co-registered PET and DW Images from an ischemic stroke patient. Figure 4(A) illustrates the main finding that the DWI lesion contains both core and penumbral tissue. Row 5 illustrates the core (CMRO2, CBF) and penumbra (OEF) voxels (*red* and *green* contours, respectively) with the DWI ROI superimposed (*black* contours). As expected, the relative proportion of penumbra was highest at the dorsal- and ventral-most regions of the middle cerebral artery territory, and lowest in its centre. In Figure 4(B) Co-registered day 30 high-resolution spoiled gradient echo sequence scan show final infarct (equivalent slices to Figure 4(A). Superimposed are the PET core defined by CMRO2 and CBF (*red*) and penumbra defined by OEF (*green*). The PET-derived ROIs were interpolated to the higher-resolution magnetic resonance image. Visual assessment confirms that, bearing in mind a 95% probabilistic threshold, the core ROI translates into infarcted tissue, whereas the penumbra ROIs (as expected) have a mixed outcome, which would fit with the documented associated clinical improvement of the patient from an NIHSS of 16 to 9. Comparing the DWI lesion ROI from Figure 4(A), it can be seen that most of it progresses to infarction, but note the variable DWI lesion intensity. In these images, the right hemisphere is on the right. Modified and reproduced with permission [60].

The introduction of MR diffusion-weighted imaging (DWI) and perfusion-weighted imaging opened a new era of stroke imaging and a second evolution of imaging technology was conceived from the PET based concept of penumbra to the MR based concept of mismatch. The term mismatch applies a two-compartment approach: the infarct core is delineated on maps of DWI intensity or the apparent diffusion co-efficient (ADC), and the area of low perfusion is delineated on maps of perfusion-weighted imaging (Figure 3) [61–63]. The volumetric difference of normal appearing tissue on DWI and hypoperfusion on perfusion-weighted imaging is termed “mismatch” between these two maps. The mismatch is considered at risk of infarct growth without reperfusion and shows characteristics of the penumbra. To support the concept it has been shown that the core volume correlates with stroke severity and predicts large parts of the final infarcted tissue and the rescue of so-called penumbral tissue correlates with clinical improvement [64–67]. Two types of study methodology have been used to validate the mismatch hypothesis: the first method has reviewed DWI/PWI magnetic resonance imaging to define imaging patterns that show irreversibly infarct tissue *vs.* salvageable tissue in the presence or absence of reperfusion. This approach utilizes large patient numbers but the longitudinal design of these studies is limited by the absence of CBF data between imaging time points [50]. The second and alternative approach that has been undertaken has compared MR findings with a “gold standard” reference method typically PET (Figure 4). Recent clinical trials using the mismatch paradigm to select patients treated with thrombolysis did not show superior outcome, further questioning the validity and utility of the paradigm for making clinical stroke management decisions [1,12,14,68]. The mismatch concept can be scrutinized by asking two broad questions: (1) Does diffusion-weighted imaging identify ischemic core; (2) Can MR mismatch reliably identify the ischemic penumbra?

### (1) Does diffusion-weighted imaging identify ischemic core?

The Apparent Diffusion Coefficient (ADC) quantifies the diffusibility of water and absolute thresholds of ADC reduction have been quoted as an absolute viability threshold in the controlled environment of animal stroke models but not humans. Clinically the timing of the ADC measurement is critical to its interpretation as it normalizes and then increases over time. It is also recognized that there is regional tissue susceptibility reflected by a variance of vulnerabilities; reduction of ADC in the striatum correlated with reduced ATP and tissue necrosis. The response of ADC upon reperfusion is an observed return of the ADC toward normal values [69,70]. There are numerous examples in the clinical literature that support that that ADC is reversible under the circumstances of prompt reperfusion, but as yet there is no agreed upon standard ADC value that predicts final infarct size. Despite potential ambiguity in interpreting DWI changes, [71,72] regions of DWI hyperintensity currently provide the best estimate of infarct core and often correspond well to regions of permanent damage.

T2-weighted imaging is sensitive to inter-parenchymal water accumulation, and increased T2 becomes apparent within several hours. T2 has become generally accepted as a reliable technique to predict an ischemic lesion beyond the acute stages. It correlates with histological measures of hemispheric swelling and the area of infarction by TTC and H&E histological sections [73]. The detection of increased intensities in T2 weighted images within first 10 hours of stroke onset represents tissue destined for final infarction and is a robust marker of final infarction demonstrated histopathologically [74]. However, again the infarct size defined by T2 is dependent on time because the maximum T2 response occurs within 48 hours, and beyond this time point dramatic changes in both size and the heterogeneity of the hyperintense T2 region occurs reflecting heterogeneous pathology in the regenerative phase of the stroke injury [74]. By acquiring different combinations of MRI signal (ADC, T2) complementary information can be gathered about the histopathological injury correlate. Reduced ADC in the presence of normal T2 may imply a less severe injury (sub lethal) consisting of compromised tissue without significant increased blood brain barrier permeability. On the contrary, the presence of an elevated T2 value may reflect more severely damaged tissue (lethal) [74–76].

### (2) Can MR mismatch reliably identify the ischemic penumbra?

Dynamic contrast perfusion-weighted imaging uses bolus tracking techniques and intravenous administration of paramagnetic contrast agents. Different curve patterns can be derived with or without deconvolution using an arterial input function obtained from large intracranial vessels such as the middle cerebral artery [77]. There are fundamental limitations with this approach. Time-to-peak (TTP), a single measure of arrival of contrast agent, does not represent the hemodynamic principle of CBF. There are other limitations. Hitherto, there is no consensus which perfusion map (TTP, relative Mean Transit Time (MTT), CBF, T_MAX_) most accurately identifies hypoperfusion and infarct growth or response to thrombolysis [78]. Also, superiority of deconvolved maps compared to non-deconconvolved maps has not shown to be superior in clinical studies [79,80].

To differentiate penumbra from benign hypoperfusion (oligemia) a visual analysis is inadequate. Numerous MRI thresholds have been used, including relative TTP, relative MTT, CBF and T_MAX_ [69,79–81]. Studies that have compared PET and MR thresholds have shown large variability of a calculated mismatch according to TTP and CBF which consistently over-estimates penumbra [80,82]. A recent study shows greater concordance of MR perfusion thresholds with PET thresholds. Increase in the TTP threshold has partially improved results [82]. One of the major problems encountered is the large inter-individual variability. For the mismatch concept to be generalizable there has to be conformity on the definition applied. Up to 49 different definitions for MR mismatch and CT penumbra have been applied in the literature to date [83]. Comparative PET and MRI studies attempted to validate the mismatch hypothesis, finding that mismatch overestimated the penumbra [60,82]. There was also no agreement on the percentage of mismatch required for therapeutic decisions. The way forward for the mismatch concept has been compromised by small, unblinded studies and the absence of data from large randomized controlled trials. An arbitrary use of a volumetric difference between diffusion lesion and perfusion lesion of 20% has been used in many studies without scientific basis [50]. This is a concern because in the clinical setting patients could be inappropriately stratified by a surrogate measure of penumbra that has not been validated. Furthermore, the mismatch phenomenon may simply be difficult to identify because the penumbra determined using a single flow threshold is not capable of representing the complexity of the physiological condition. Despite these issues, proponents note that most of these studies were performed with early non-quantitative methods. More recent trials suggest there may be utility for penumbral selection when using automated, standardized, and quantitative techniques [12,84].

## 5. Imaging of the Neurovascular Unit Dysfunction (NVU)

It has been acknowledged that understanding injury at the NVU following ischemia reperfusion is fundamental to therapeutic advancement for stroke. To date, tissue plasminogen activator (tPA) is an effective yet vastly underused treatment for acute ischemic stroke associated with several limitations to its use. First, the six-fold increase in morbidity and four-fold increase in fatality related to tPA-induced hemorrhagic transformation have deterred physician use of the therapy [85]. Second, current guidelines on thrombolysis post-stroke with tissue plasminogen activator exclude its use when time of onset is unknown [13]. Most patients do not respond to thrombolysis and mechanisms for such blunted response are not well characterized. Current MRI criteria using diffusion and perfusion imaging have concentrated attempts to define salvageable brain tissue. The relationship between stroke severity, the early inflammatory responses, BBB permeability, risk of hemorrhagic transformation, and incomplete microvascular reperfusion has largely been underdeveloped.

While our understanding of molecular and biochemical responses that ensue following cerebral ischaemia in individual cell types (neurons, glia, endothelium) has grown appreciably there is a demand to understand these processes together at the microvascular level. MRI has allowed us to study animals over time, increasing our understanding of the temporal and spatial evolution of ischemic brain injury, while also reducing the large number of animals that are required for traditional histological experiments. In addition to making assessments of what injury might be reversible *vs.* irreversible during ischemia, the use of MR paramagnetic contrast agents allows complimentary assessments of vascular flow, tissue perfusion and BBB integrity. Relevant mechanisms of injury to tissue viability and the ischemic penumbra that can be imaged are the early inflammatory responses to ischemia in the microvasculature, BBB dysfunction and CBF/neuronal changes (already discussed above).

The potential of imaging molecular and cellular events that are critically involved in stroke pathophysiology and infarct evolution at the neurovascular level has capitalized on advancements in contrast agent design and synthesis. Cellular labeling and molecular targeting with contrast agents may enable both detection and quantification of neuroinflammatory processes such as infiltration of leukocytes and upregulation of markers of endothelial activation [86,87]. These developments have promoted MRI based detection of inflammatory cells and molecular markers in pre-clinical studies, which has recently led to the parallel studies in human subjects [88,89]. MR imaging has several advantages over other commonly used molecular imaging techniques such as nuclear [90], optical [91,92], and positron emission tomography (PET), including a lack of radioactivity, high spatial anatomical resolution [93] and clinical accessibility. The intrinsic contrast can be augmented by the use of targeted contrast agents in both the experimental and clinical setting [90].

In the pursuit of determining specific disease process, the evolving field of molecular imaging requires the development of novel classes of MR detectable agents with improved image contrast. The aim of Magnetic Resonance Molecular Imaging is to provide an anatomical visualization of specific molecular processes ideally with high temporal and spatial resolution. There are some basic imaging requirements of Magnetic Resonance Molecular Imaging that should be considered: (1) the imaging target should be well-characterized with respect to its anatomical localization, the time course and the extent of expression; (2) the targeting ligand should be able to access the target, have high binding affinity and a pharmacokinetic profile suited for the needs of the imaging: (3) the signal should unambiguously be a consequence of ligand-target binding, representing specific binding.

**Figure 5.**
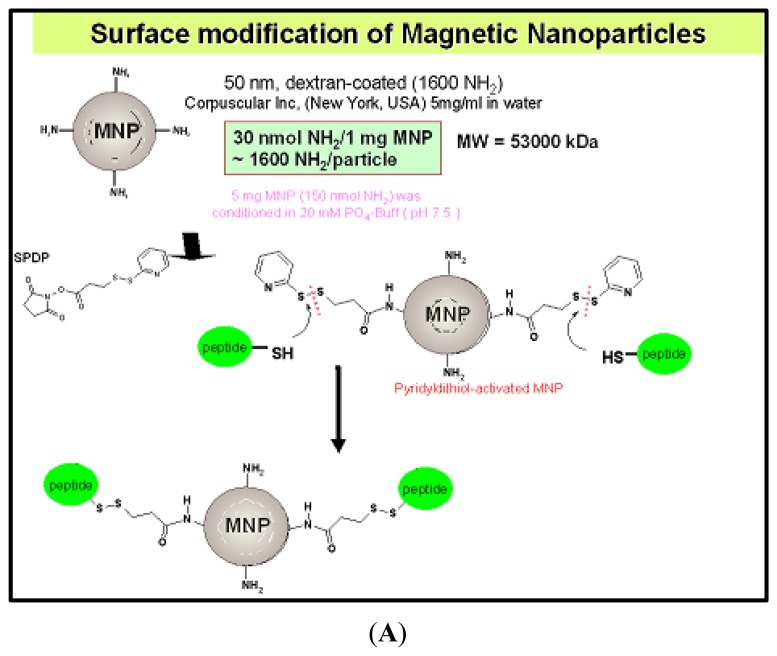

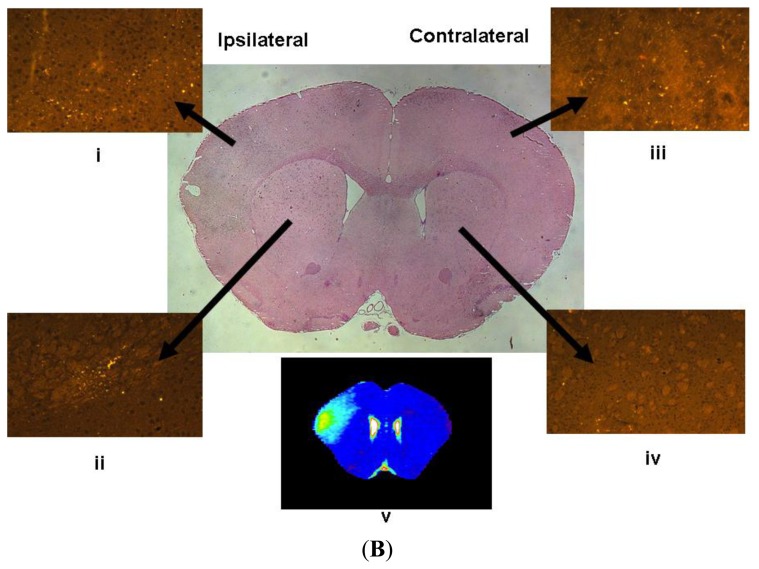
Iron-Based Magnetic Nanoparticles (MNP). **(A)** The paramagnetic core of the modified MNP consists of an iron oxide particle 10 nm in diameter and possessing a Fe_2_O_3_:FeO ratio of 2:1. This iron oxide core is enclosed within a dextran coat, thus providing numerous sites for surface modification. This allows conjugation of the MNP to an appropriate ligand of a specific biomolecular target. In this specific case, the MNP is conjugated to a unique dual peptide construct consisting of a 15-residue domain with high affinity for P-selectin linked to a second 24-residue thrombin-binding domain. The P-selectin binding domain consists of the primary amino acid sequence LVSVLDLEPLDAAWL and was discovered through the use of phage display technology. **(B)** Hematoxylin and eosin coronal section (7 micron slice thickness, bregma +1.00 mm) 26 hours after ischemia/reperfusion and two hours after MNP-PBPl injection. (i) Ipsilateral cortex, Cy5.5-anti-P-selectin-IgG, (ii) Ipsilateral striatum, (iii) Contralateral cortex, (iv) Contralateral striatum, (v) T2 map coronal slice (bregma +1.18 mm) showing the infarct as a light blue area with yellow-green center [87].

Currently two major classes of contrast agents exist: paramagnetic (gadolinium based); and super-paramagnetic agents [94]. The paramagnetic contrast agents shorten both T1 and T2 relaxation, but preferentially T1. They create a hyperintense contrast on conventional T1 weighted spin echo sequences. The second class of agent is based on superparamagnetic iron oxide (SPIO) particles. In a magnetic field the net magnetic moment is several orders of magnitude greater than the paramagnetic agents (Figure 5). This creates extremely large microscopic field gradients for dephasing nearby protons [95,96], and that is accompanied by a substantial decrease in T2. Beyond conventional MRI sequences, T2 imaging causes further shortening of the relaxation properties of the tissue.

**Figure 6.**
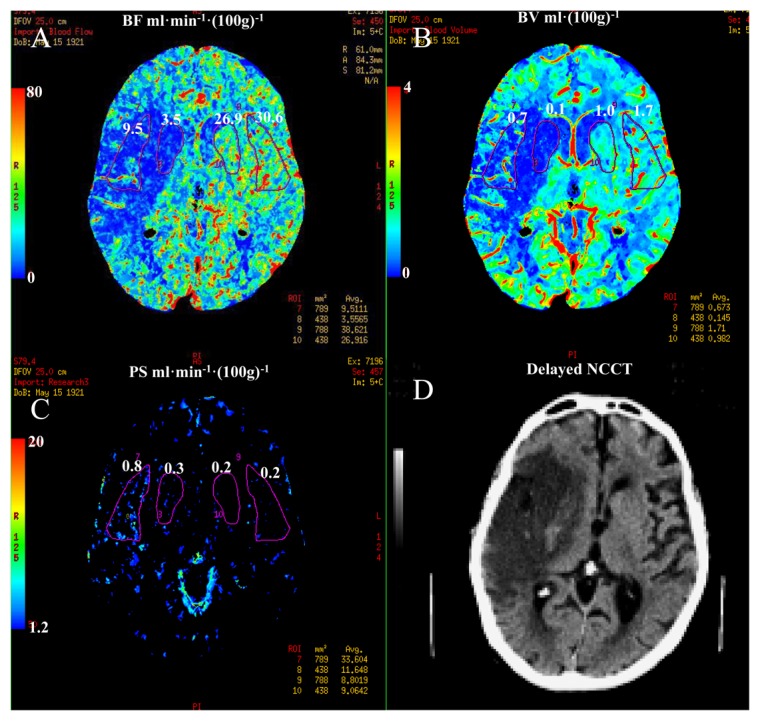
Permeability surface (PS) area product map from a patient with a large area of contrast leakage into the ischemic region: (**A**) CBF map of a 5 mm thick brain slice from the admission CTP study displayed with a color scale from 0 (dark blue) to 150 (red) mL·min^−1^·(100 g)^−1^ (**B**) of pixels with CBF less than 25 mL·min^−1^·100 g)^−1^; (**C**) corresponding PS map to CBF map in (a) showing the superimposed ischemic ROI and the mirrored ROI in the contralateral hemisphere. PS values in the ischemic and mirrored ROI were 0.40 and 0.122 mL·min^−1^·(100 g)^−1^ respectively; (**D**) corresponding delayed non-contrast CT scan of the same slice showing infarct and hemorrhagic transformation (red arrow). Courtesy of Chris d'Esterre, Richard Aviv and Ting-Yim Lee (University of Western Ontario).

Techniques capable of characterizing BBB integrity may prove particularly valuable because there is a credible association between BBB disruption and risk of hemorrhagic transformation with thrombolytic therapy. For the assessments of BBB permeability paramagnetic contrast agent is administered intravenously using a bolus injection. With an intact blood brain barrier the contrast agent passes through the microvasculature of the brain, being contained to the intravascular space. During stroke there are areas of the brain that are associated with BBB breakdown and it is in these areas that the contrast agent can extravasate into the interstitium. There are two methods of detecting BBB breakdown: (1) static T1-weighted MR imaging; and, (2) dynamic contrast enhanced MRI (DCE MRI) [97]. Post contrast T1-weighted MRI is specific for BBB breakdown implying that it is not visualized when the BBB is not dysfunctional but its limitations relate to the infrequency of its detection during the early period after symptom onset and it is relatively insensitive to predicting hemorrhagic transformation (sensitivity 39%) [98,99]. Therefore, post contrast T1-weighted MRI does not have sufficient diagnostic accuracy for routine clinical decision-making. The low sensitivity of post contrast T1 MRI may be partially explained by the failure of contrast arriving to the ischemic during either microvascular or large artery occlusion. The addition of continuous low dose infusion of contrast to the initial bolus dose could improve the sensitivity of post contrast T1-weighted MRI [100]. The downside of this technique is that it requires a long image acquisition, which may be impractical in the context of acute ischemic stroke. The availability, speed and accessibility of CT are the main reasons that CT remains the diagnostic procedure of choice for the treatment of stroke with thrombolysis. CT is capable of generating physiological information about the penumbra. This can be achieved by a 40–50 s DCE or CT perfusion examination. By extending the acquisition time information about BBB integrity or permeability can be obtained (Figure 6) [101].

More research is required in this area but it is hypothesized that dynamic contrast MRI or CT may be useful in the prediction of hemorrhagic transformation in acute ischemic stroke. It has been proposed that measurements of BBB permeability in acute stroke may aid in selecting acute ischemic stroke patients for treatment based on imaging criteria of BBB integrity, avoiding the restrictive eligibility criteria mandated by the time window paradigm. Knight *et al.* showed that DCE MRI in a rat model of progressive parenchymal enhancement was highly correlated with the presence of hemorrhagic transformation [102,103]. The main potential drawback is that BBB permeability following ischemic stroke is physiologically a highly dynamic process, and therefore, the imaging at one time point may only provide “snapshot” may not be represent the disease process at a later time point[97]. Such techniques have been performed in human subjects, but it is as yet too early to formalize a permeability threshold facilitating treatment allocation.

## 6. Conclusions/Outlook

Neuroimaging has been fundamental to translating pathophysiological concepts to clinical trials. The target for stroke therapy remains the ischemic penumbra, *i.e.*, brain with reduced blood flow at risk of infarction. PET is considered to be the gold standard for CBF quantification with the additional parameter of tissue viability in terms of maintained rates of cerebral oxygen metabolism. MR diffusion and perfusion-weighted imaging have been used to translate the imaging concept of penumbra to one of diffusion-perfusion mismatch. This conceptual framework of penumbra is not absolute and controversies remain regarding whether diffusion can correctly identify infarct core and perfusion-weighted imaging can identify tissue at risk outside the DWI lesion. Other problems exist regarding consensus over definitions of penumbra, the consistency of post-processing software used between research groups, and, mismatch has not been shown to be a reliable surrogate to target patient populations in prospective studies. There remain several voids clinically of what we understand of reversible and irreversible injury from the imaging technology and what we understand of the disease at the microscopic level, specifically our concept of the neurovascular unit, its cellular interrelationships and clinical importance in terms of its dependence of tissue viability on blood flow return. The combination of neuroimaging modalities that include biomarkers of both blood flow, metabolism and blood brain barrier breakdown along with molecular imaging markers of cell injury may clearly delineate reversible *vs.* irreversible and potentially salvageable tissue in the penumbra. This will also expand our perspective that the identification of tissue at risk is only one of many predictors of therapeutic success. The treatment related risks have to be considered and limit therapeutic options for instance related to intracerebral hemorrhage with thrombolysis. Therefore the mismatch concept has to be extended to the assessment of the regional integrity of the neurovascular unit to estimate the response and risk associated with treatment. Under certain therapeutic situations the response of ischemic tissue to reperfusion is likely to exacerbate injury and therefore BBB integrity may provide a useful surrogate to assess ability to rescue tissue. A more physiological based approach complementary to our current understanding of the biological mechanisms, should allow decision processes that alleviate our dependence on the current time based treatment window to an imaging based treatment window [104,105]. Future directions of MRI will refine the mismatch concept by providing complimentary pathophysiological information to current compartmental paradigms of ischemic viability (DWI) and perfusion (PWI) with imaging information related to CBF, BBB permeability, early inflammation, oedema formation in the early acute phase, and further refinements developed (e.g., with microglial or cell death molecular imaging markers) and enhanced BBB permeability markers).
